# Immediate Dentin Sealing for Adhesive Cementation of Indirect Restorations: A Systematic Review and Meta-Analysis

**DOI:** 10.3390/gels8030175

**Published:** 2022-03-11

**Authors:** Louis Hardan, Walter Devoto, Rim Bourgi, Carlos Enrique Cuevas-Suárez, Monika Lukomska-Szymanska, Miguel Ángel Fernández-Barrera, Elizabeth Cornejo-Ríos, Paulo Monteiro, Maciej Zarow, Natalia Jakubowicz, Davide Mancino, Youssef Haikel, Naji Kharouf

**Affiliations:** 1 Department of Restorative Dentistry, School of Dentistry, Saint-Joseph University, Beirut 1107 2180, Lebanon; louis.hardan@usj.edu.lb (L.H.); rim.bourgi@net.usj.edu.lb (R.B.); 2 Independent Researcher, 16030 Sestri Levante, Italy; walter@walterdevoto.com; 3 Dental Materials Laboratory, Academic Area of Dentistry, Autonomous University of Hidalgo State, Circuito Ex Hacienda La Concepción S/N, San Agustín Tlaxiaca 42160, Hidalgo, Mexico; miguel_fernandez10334@uaeh.edu.mx (M.Á.F.-B.); elizabeth_cornejo@uaeh.edu.mx (E.C.-R.); 4 Department of Restorative Dentistry, Medical University of Lodz, 251 Pomorska St., 92-213 Lodz, Poland; monika.lukomska-szymanska@umed.lodz.pl; 5 Clinical Research Unit (CRU), Centro de Investigação Interdisciplinar Egas Moniz (CiiEM), Egas Moniz, CRL, Monte de Caparica, 2829-511 Caparica, Portugal; paulojorgemonteiro@yahoo.ca; 6 Private Practice, “NZOZ SPS Dentist” Dental Clinic and Postgraduate Course Centre, pl. Inwalidow 7/5, 30-033 Cracow, Poland; dentist@dentist.com.pl (M.Z.); nljakubowicz@gmail.com (N.J.); 7 Department of Biomaterials and Bioengineering, INSERM UMR_S 1121, Biomaterials and Bioengineering, 67000 Strasbourg, France; endodontiefrancaise@outlook.com (D.M.); youssef.haikel@unistra.fr (Y.H.); 8 Department of Endodontics, Faculty of Dental Medicine, Strasbourg University, 67000 Strasbourg, France

**Keywords:** adhesive, bond performance, delayed dentin sealing, dentin bonding agent, immediate dentin sealing

## Abstract

Immediate dentin sealing (IDS) involves applying an adhesive system to dentin directly after tooth preparation, before impression. This was considered an alternate to delayed dentin sealing (DDS), a technique in which hybridization is performed following the provisional phase and just before the indirect restoration luting procedure. This study aimed to compare the bond strength of restorations to dentin of the IDS and the DDS techniques throughout a systematic review and meta-analysis. The following PICOS framework was used: population, indirect restorations; intervention, IDS; control, DDS; outcomes, bond strength; and study design, in vitro studies. PubMed (MedLine), The Cochrane Library, ISI Web of Science, Scielo, Scopus, and Embase were screened up to January 2022 by two reviewers (L.H. and R.B.). In vitro papers studying the bond strength to human dentin of the IDS technique compared to the DDS technique were considered. Meta-analyses were carried out by using a software program (Review Manager v5.4.1; The Cochrane Collaboration). Comparisons were made by considering the adhesive used for bonding (two-step etch-and-rinse, three step etch-and-rinse, one-step self-etch, two-step self-etch, and universal adhesives). A total of 3717 papers were retrieved in all databases. After full-text assessment, 22 potentially eligible studies were examined for qualitative analysis, leaving a total of 21 articles for the meta-analysis. For the immediate bond strength, regardless of the adhesive strategy used, the IDS technique improved the bond strength of restorations to the dentin (*p* < 0.001). Taking into account the subgroup analysis, it seems that the use of the IDS technique with a two-step etch-and-rinse or a one-step self-etch adhesive system does not represent any advantage over the DDS technique (*p* = 0.07, *p* = 0.15). On the other hand, for the aged bond strength, regardless of the adhesive strategy used, the IDS technique improved the bond strength of restorations to the dentin (*p* = 0.001). The subgroups analysis shows that this improvement is observed only when a three-step etch-and-rinse adhesive system (*p* < 0.001) or when a combination of an adhesive system plus a layer of flowable resin (*p* = 0.01) is used. The in vitro evidence suggests that the use of the IDS technique improves the bond strength of dentin to resin-based restorations regardless of the adhesive strategy used. The use of a three-step etch-and-rinse adhesive system or the combination of an adhesive system plus a layer of flowable resin seems to considerably enhance the bond strength in the long term.

## 1. Introduction

Direct resin composite restorations are most preferred by patients as adequate not only for anterior teeth, but also for posterior teeth. The use of this kind of restoration has increased and a lot of improvements in resin materials formulations and clinical techniques have been developed in recent years [1,2]. Despite this, the performing of large restorations including the proximal region presents challenges for direct resin composite restorations, such as the recovery of an adequate proximal contacts, anatomical form, wear resistance, and marginal adaptation [3,4]. Polymeric or ceramic-based indirect restorations are an effective treatment to resolve these issues. In fact, partial adhesive indirect restorations present multiple advantages when compared with direct restorations, including improved anatomic shape, contour, esthetics, and fracture resistance [5].

Conventional indirect restoration involves a series of complicated procedural steps. These restorations are laboratory fabricated and require at least two appointments: one for preparation and impression/model fabrications, and one for luting [6]. In the first appointment, an impression is immediately taken after tooth preparation, followed by luting a temporary restoration. In the second appointment, once the indirect restoration is fabricated, the temporary restoration is removed, then a bonding agent is applied to the dental substrate; next, for the adhesive luting procedure, a resin luting agent is applied [7]. In this technique, known as delayed dentin sealing (DDS), dentin hybridization is performed following the provisional restorations and just previously to the indirect restoration luting process. This technique has particular drawbacks, as residual temporary cement might persist on the dental surface, and some cement constituents perhaps infiltrate the dental surface [8]. In this fashion, the definitive restoration is commonly not bonded to freshly prepared dentin but quite bonded to contaminated dentin, which could result in hybridization failure and lessened bond strength [9].

In order to prevent this, the immediate dentin sealing (IDS) method has been advised [10,11]. In this procedure, an adhesive system is applied directly to the fresh cut dentin preceding the placement of the provisional phase. IDS demands hybridization of the freshly dentinal surface closely after preparing the tooth and before the luting processes [12,13]. This technique provides adhesion to a freshly cut and uncontaminated dentin, which is ideal for bonding [14,15]. In addition, the technique prevents the bacterial invasion and dentin sensitivity during the provisional phase [16]. Another advantage is that the thickness of the dentin bonding agent is considered before the tooth preparation impression [17].

Theoretically, the method might be performed using any adhesive system, leading to enhanced bond strength when compared to DDS; nevertheless, this was not systematically reviewed. On the other hand, the general procedure proposes the use of two-step self-etch or three-step etch-and-rinse adhesive systems as well [9]; besides this, with recent developments in adhesive dentistry, clinicians have a wide range of adhesive materials available, including two-step etch-and-rinse, three step etch-and-rinse, one-step self-etch, two-step self-etch, and universal adhesives, and the question as to whether all adhesive systems currently available could have an optimal performance for IDS still remains. Considering this, the objective of this study was to systematically review the literature to compare the bond strength of restorations to dentin between the IDS and the DDS techniques. The null hypothesis of the study was that IDS or DDS techniques would provide the same bond strength.

## 2. Results and Discussion

**Figure 1:**
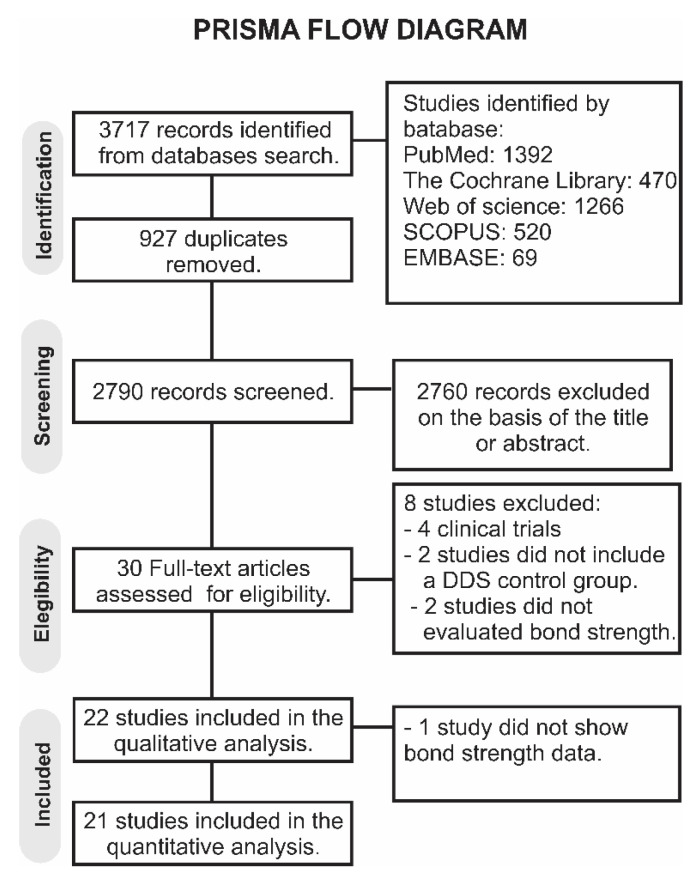
PRISMA flow diagram of the study.

A total of 3717 papers were retrieved in all databases. A flowchart designating the study selection process according to the PRISMA Statement is presented in Figure 1. The literature review retrieved 2790 papers for the initial examination after eliminating the duplicates. Afterward, 2760 studies were excluded following reviewing the titles and abstracts, leaving a total of 30 articles to be assessed by full-text evaluation. From these, 8 studies were not considered for the qualitative analysis for various reasons: 4 were clinical studies [18,19,20,21], 2 studies did not evaluate bond strength [22,23], and in 2 studies, there was not a control group using DDS [24,25]. Thus, a total of 22 studies were considered in the qualitative analysis. One study was excluded from the quantitative analysis because data on bond strength were not available [26], giving 21 studies considered for the meta-analysis [12,27,28,29,30,31,32,33,34,35,36,37,38,39,40,41,42,43,44,45,46].

The characteristics of the studies included in this review are summarized in Table 1.

The included studies tested several types of adhesives for IDS, including two-step etch-and-rinse, three-step etch-and-rinse, one-step self-etch, two-step self-etch, and universal adhesives. The bond strength was evaluated using the micro-tensile or the shear bond strength tests.

**Figure 2:**
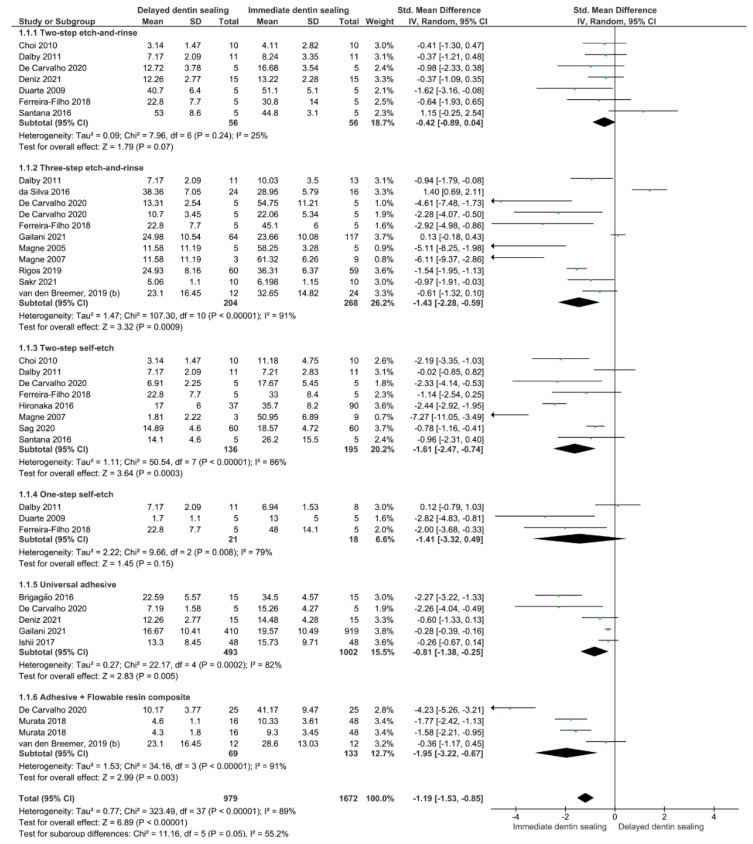
Forest plot of the immediate bond strength comparison between the delayed and immediate dentin sealing techniques according to the adhesive used.

A meta-analysis of the 21 in vitro studies was conducted [12,27,28,29,30,31,32,33,34,35,36,37,38,39,40,41,42,43,44,45,46], and separate analyses for immediate and aged bond strength were performed. Separate subgroups were analyzed for each aging time considering the type of adhesive used. For the immediate bond strength (Figure 2), regardless of the adhesive strategy used, the IDS technique improved the bond strength of restorations to the dentin (*p* < 0.001). Taking into account the subgroup analysis, it seems that the use of the IDS technique with a two-step etch-and-rinse or a one-step self-etch adhesive system does not represent any advantage over the DDS technique (*p* = 0.07, *p* = 0.15).

**Figure 3:**
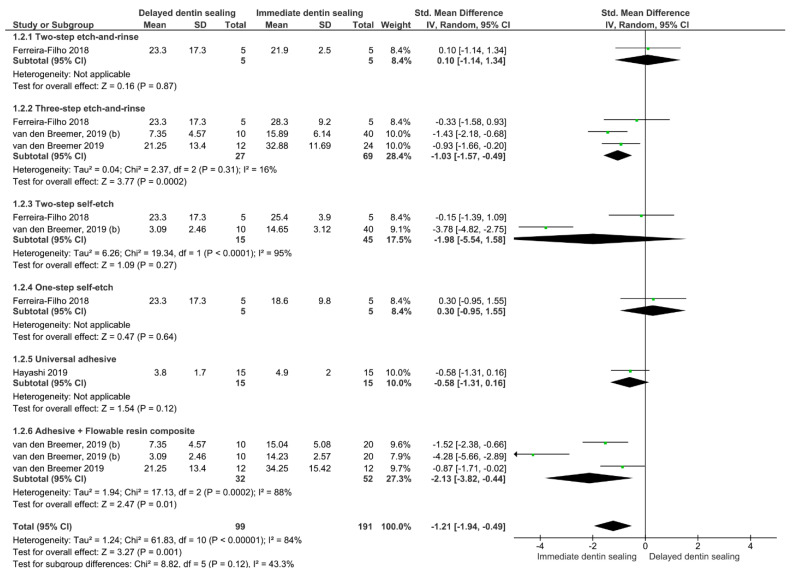
Forest plot of the aged bond strength comparison between the delayed and immediate dentin sealing techniques according to the adhesive used.

On the other hand, for the aged bond strength (Figure 3), regardless of the adhesive strategy used, the IDS technique improved the bond strength of restorations to the dentin (*p* = 0.001). The subgroups analysis shows that this improvement is observed only when a three-step etch-and-rinse adhesive system (*p* < 0.001) or when a combination of an adhesive system plus a layer of flowable resin (*p* = 0.01) is used.

Studying the parameters of methodological quality assessment, most of the manuscripts involved were counted with a medium risk of bias (Table 2); though several studies analyzed failed to account for the sample size calculation, single operator, and operator blinded parameters.

A systematic review and meta-analysis was achieved to compare the bond strength of restorations to dentin between the IDS and the DDS techniques. For the immediate and aged bond strength, regardless of the adhesive strategy used, the IDS technique improved the bond strength of restorations to the dentin. Considering this, the null hypothesis proposed in this research was rejected.

Th immediate bond strength was improved when the IDS strategy was used. It seems that the use of the IDS technique with a two-step etch-and-rinse or a one-step self-etch adhesive system does not represent any advantage over the DDS technique. One should bear in mind that applying bonding agents to dentin after preparing dental cavity might increase the bond strength [12]. Furthermore, the resulting hybrid layer (HL) was stable, especially when filled adhesive was applied for IDS [14]. After tooth preparation, IDS reduces the formation of microleakage between dentin surface and restoration, thus lessening bacterial contamination and pulpal sensitivity [15,47]. Consequently, better adaptation of the restoration to the cavity surface could be expected [13]. Nevertheless, by applying the traditional DDS technique, a gap could be formed between the resin and dentin [48].

During the conventional bonding process, bonding agents were applied when indirect restorations were seated to the tooth structure in cementation [28]. In an attempt to avoid the incomplete seating of dental restorations, clinically, it is advisable to retain the adhesive resin unpolymerized before the placement of laminate veneer, for example [49]. This could be explained by the fact that polymerized dentin adhesives presented a thickness varying from 60–80 μm to 200–300 μm depending on the tooth surface structure [50,51], though a thickness of fewer than 40 μm was acclaimed before indirect restoration placement [51]. Nonetheless, it is stated that curing resin cement and dentin adhesive agent individually in order displayed superior bond strength than curing both concurrently [50,52]. This comes from the idea that unpolymerized resin–dentin HL collapses when placing the restoration [48,53]. In this manner, efforts were directed towards the optimization of the application of bonding agents [54,55]. This could be possible by applying the adhesive system after preparing the tooth and before taking the impression [56]; thus, this technique is also known as IDS [12]. Further, to demarcate the conventional dentin bonding agent from IDS, it is called DDS [28].

It is important to define the interdiffusion layer, also called HL, which forms between the adhesive resin and the demineralized dentin, hence, playing a noteworthy role on the retention of the restoration [57,58,59]. This HL was not stable with time due to the degradation process [60]. Once using any type of adhesive systems, uninfiltrated and demineralized dentin structure with exposed collagen networks could be revealed in the HL [61,62]. Bonding systems that possess a hydrophobic resin coating the primer layer play an essential part in lessening dentin permeability [63]. It has been suggested that adequate sealing could be possible when using the IDS with a two-step etch-and-rinse or a one-step self-etch adhesive system, but the complete elimination of marginal microleakage could not be possible [64]. This was unfortunately elucidated by the formation of some porosities within the HL [65]. In addition, these adhesive systems were subjected to outward fluid flow [66]. The inward and outward fluid moves generate the movement of water trees described by Tay et al., which can provide to the degradation of the resin-bonded interfaces [67]. This phenomenon could support the finding of this study as these adhesives do not represent any advantage over the DDS technique. There may also be issues with the one-step self-etching adhesives being too hydrophilic, contributing to the increased permeability of the HL, and they may be subject to hydrolysis and chemical decomposition; next, resulting in an incompatibility with chemical or dual-cure composite luting agents [68,69].

Aged bond strength was improved when the IDS strategy was used and this improvement was only detected when a three-step etch-and-rinse adhesive system was used, or when a combination of an adhesive system plus a layer of flowable resin was used. Normally, adequate resin–dentin bonding is immediately reached, yet diminished bonding effectiveness occurs with time due to interfaces degradation [70]. The IDS approach was considered to resist the mechanical loading and heat for a longer period of time, moreover, improved the marginal adaptation between dentin and the restoration [28]. This matches with the discovery of this manuscript, as aged bond strength was perfected with the IDS technique.

Three-step etch-and-rinse adhesive systems were very effective when used correctly, and were the most versatile of all adhesive generations, as they can be used for virtually any bonding protocol (direct, indirect) or photopolymerization (self-cure, dual-cure). These systems were still the standards by which new systems were judged [69,71]. In fact, higher bond strength was achieved by using these systems in an IDS strategy, like the use of Optibond FL (Kerr; Orange, CA, USA), a filled adhesive resin with an ability to form a uniform film thickness of approximately 88 μm. This makes the filled adhesive more suitable for IDS than unfilled adhesive [72]. The filled adhesive was also perceptibly noticeable, a fact that made the assessment of the dentin bonding agents during placement simpler, as well as after surface cleaning preceding the definitive cementation [13,73].

A crucial component in IDS is the elaboration of an effective resin-to-resin bond between the presented resin coating and the new luting resin. This could be achievable with the use of older adhesive systems (three-step etch-and-rinse adhesive systems) showing their performance at the bonded interface level, in terms of stability, bond strength, and aging [74]. This could be in agreement with the results obtained in this review.

The use of a combination of an adhesive system and a layer of flowable resin seems to improve the adhesive strength [75]. This appears exceptionally principal to the performance of simplified adhesive systems to safeguard the thin bonding interface from oxygen inhibition and preserve IDS coat through the cleaning of the preparation [73]. By doing so, a thick adhesive layer was created. A thick IDS helps in eliminating undercuts and provides a smooth preparation [47]. However, a thin IDS layer is more susceptible when using silica coating, and the dentin might become re-exposed [28].

Additionally, by applying a flowable composite on top of a low film-thickness adhesive, thicker flowable composite may serve as an internal stress absorber, maintaining the integrity of the adhesive interface over time. This may be specifically beneficial in deep proximal boxes of posterior restorations, which will lead to better marginal adaptation at the axial box and critical cervical margins [45,76,77]. Moreover, a previous study [78] showed the importance of protecting the HL with a low stress bulk-fill flowable composite after aging in an oral environment in terms of perfect adaptation and seal. This potential explanation could confirm the long-term performance of applying such a strategy in this research.

The methodological quality assessment showed that most articles involved were categorized with a medium risk of bias, which identifies that the value of the discovery studied can be high. In this manner, it could be highlighted that the sample size calculation, the single operator, and the operator blinding were not designated in most of the articles examined. In addition, failure to describe these factors might increase the probability of finding bias and implementation [79]. In addition, it is worth mentioning that most of the comparisons performed presented a moderate heterogenicity (I^2^ ≥ 43.3%), and the reasons for these values of heterogenicity could include differences in the methodological design, like the number of samples tested and bond strength tests used, and differences in the variability outcomes.

From this systematic review and meta-analysis, in vitro evidence was evaluated with regards to comparing by searching in the literature the bond strength of restorations to dentinal surface between two techniques: the IDS and the DDS techniques. The findings of this study should be carefully taken as in clinical practice, a wet environment, masticatory stresses, and pH trigger a rapid degradation of the adhesive–dentin interfaces. This in vitro study offers important information when considering new techniques such as IDS to increase bond to dental tissues.; though they have limitations and do not replace clinical trials.

A recent systematic review and meta-analysis explored if the IDS technique influences postoperative sensitivity in teeth restored with indirect restorations [80] and concluded that there is low-certainty evidence obtained from clinical trials demonstrating that IDS does not reduce postoperative sensitivity in teeth restored with indirect restorations. The low evidence is explained by the fact that only two clinical trials were included in the review, and there were differences in the adhesive strategy and luting agent type used. Summarizing the in vitro evidence collected in this tested review, it should be highlighted that the IDS technique lacks standardization. By analyzing the data presented in this meta-analysis, the use of a three-step etch-and-rinse adhesive followed by a layer of flowable resin could be recommended to ensure the maximum retention of the indirect restoration.

Further examination should be directed, remarkably randomized controlled clinical trials, with the purpose of attaining a better understanding of the performance of IDS technique in the clinical success of indirect restorations to dentin substrate. Next, additional studies measuring cyclic loading and long period effectiveness of IDS should be conducted. Future research is required to investigate more dental adhesives and flowable composite resins to reveal the comparison between diverse materials. It should be also recommended to perform studies with more standardized methods in an effort to reduce the heterogeneity between the studies focusing on this topic and also to establish the ideal protocol for cementing indirect restorations.

## 3. Conclusions

The in vitro evidence suggest that the use of the IDS technique improves the bond strength of dentin to resin-based restorations regardless of the adhesive strategy used. The use of a three-step etch-and-rinse adhesive system or the combination of an adhesive system plus a layer of flowable resin seems to considerably enhance the bond strength in the long term.

## 4. Materials and Methods

This systematic review and meta-analysis was performed following the PRISMA 2020 statement [81]. The registration protocol was carried out in the Open Science Framework with the registration number 0000-0002-2759-8984. The following PICOS framework was used: population, indirect restorations; intervention, IDS; control, DDS; outcomes, bond strength; and study design, in vitro studies. The research question was: “Does the use of the IDS technique improve the bonding performance of restorations?”

### 4.1. Literature Search

The literature search was independently conducted by two reviewers (R.B. and L.H.) until 20 January 2022. Six electronic databases were selected to recognize manuscripts that might be included: PubMed (MedLine), The Cochrane Library, ISI Web of Science, Scielo, Scopus, and Embase. The search strategy and keywords used in PubMed are listed in Table 3. The full search strategy for The Cochrane Library, ISI Web of Science and Scielo, Scopus, and EMBASE databases is presented as supplementary material (Tables S1–S4). The reviewers also hand-searched the reference lists of involved manuscripts for identification of supplementary papers. After the initial screening, all articles were introduced into Mendeley Desktop 1.17.11 software (Glyph & Cog, LLC, London, UK) to exclude duplicates.

### 4.2. Study Selection

Two independent reviewers (C.E.C.-S. and R.B.) assessed the titles and abstracts of all the studies. Manuscripts for full-text review were chosen agreeing to the following eligibility criteria: (1) evaluated the bond strength to human dentin of the IDS technique compared to the DDS technique; (2) included a control group with the use of the DDS technique for indirect restorations; (3) published in the English, Spanish, or Portuguese language. Case series, case reports, pilot studies, and reviews were excluded. Full copies of all the possibly appropriate papers were examined. Those that seemed to gather the inclusion criteria or had inadequate data in the title and abstract to make a clear decision were selected for full evaluation. The full-text manuscripts were considered in duplicate by two independent review authors. Any disagreement or discrepancy regarding the eligibility of the included manuscripts was resolved and decided through consensus and agreement by a third reviewer (L.H.). Only papers that satisfied all the eligibility criteria enumerated were included for review.

### 4.3. Data Extraction

Data of relevance from the studies involved were extracted using Microsoft Office Excel 2019 sheets (Microsoft Corporation, Redmond, WA, USA). These data comprised the study and year of publication, the type of tooth, the IDS, the type of cement, the type of restoration, the aging procedures, and the bond strength test used. The corresponding authors of the included studies were connected twice via e-mail to retrieve the lack of information, if any data were somewhat missing. If the investigators did not response within 2 weeks of the first communication, the missing information was not included.

### 4.4. Quality Assessment

The methodological quality of each involved manuscript was performed by two independent authors (L.H. and R.B.), by assessing the description of the following parameters: specimen randomization, single-operator protocol implementation, operator blinded, presence of a control group, standardized samples, failure mode, manufacturer’s instructions, and the sample size calculation [82]. If the reviewers stated the parameter, the study received a “YES” for that specific parameter. In the case of missing data, the parameter received a “NO.” The risk of bias was classified regarding to the sum of “YES” answers received: 1 to 3 indicated a high bias, 4 to 6 medium, and 7 to 8 indicated a low risk of bias. Through risk of bias assessment, any differences between the reviewers were resolved through conversation by accessing a third researcher (C.E.C.-S.).

### 4.5. Statistical Analysis

Meta-analyses were performed using Review Manager v5.4.1 (The Cochrane Collaboration) software program. The comparisons were performed using the random-effects model, and the standardized mean difference between the bond strength values obtained using the DDS or the IDS technique. Subgroups comparisons were made depending on the adhesive used for bonding (two-step etch-and-rinse, three step etch-and-rinse, one-step self-etch, two-step self-etch, and universal adhesives). In studies where several experimental groups were compared against the same control group, data for the experimental groups (mean, standard deviation, and sample size) were combined for the meta-analysis. Immediate and long-term bond strength data were analyzed separately. Statistical heterogeneity among studies was assessed using the Cochran Q test and the I2 test.

## Figures and Tables

**Table 1:** Main characteristics of the in vitro studies included in the review.

Study (Year)	Type of Tooth	IDS Technique	Type of Restoration	Aging Procedures	Bond Strength Test Used
Brigagão et al., 2016.	Human third molar	Application of an universal adhesive system in self-etch or etch-and-rinse mode. Temporary restoration. Cleaning with rotary brush. Application of an universal adhesive system.	Composite resin blocks luted with conventional or self-adhesive resin cement.	Distilled water at 37 °C for 7 days.	Micro-tensile bond strength (μTBS)
Choi et al., 2010.	Human molars	Application of a two-step self-etch or a two-step total-etch adhesive system.	Porcelain specimens luted with resin cement.	Distilled water at 37 °C for 24 h.	Shear bond strength (SBS)
Da Silva et al., 2016.	Human molars	Application of a three-step total-etch adhesive. Temporary restoration. Application of a three-step total-etch adhesive.	Composite resin.	Distilled water at 37 °C for 24 h.	μTBS
Dalby et al., 2011.	Human third molars	Application of a three-step total-etch, two-step self-etch adhesive system, two-step total-etch, or a one-step self-etch adhesive system.	Heat-pressed leucite reinforced glass ceramic luted with resin cement.	Distilled water at room temperature for one week.	SBS
De Carvalho et al., 2020.	Human third molars	Application of a three-step total-etch, two-step total-etch, two-step self-etch adhesive system, or a universal adhesive system. Application of an additional flowable resin coating. Provisionalization. Preparation cleaning with 50 μm aluminum oxide airborne-particle (5 s at 1.5 cm and 2 bar) and phosphoric acid (15 s, rinsed and dried) and covered with a layer of adhesive resin.	Resin composite.	Distilled water at room temperature for at least 24 h.	μTBS
Deniz et al., 2021.	Human molar	Application of a two-step total-etch or a universal adhesive system. Application of a universal adhesive system (Single Bond Universal, 3M ESPE).	Self-adhesive resin resin cement.	Distilled water at 37 °C for 24 h	SBS
Duarte et al., 2009.	Human third molars	Application of a two-step total-etch or a one-step self-etch. Temporary restoration. Surface cleaning with pumice and water. Acid etching with 35% phosphoric acid. Application of 2–3 layers of Adper Single Bond or Adper Prompt L-Pop.	Ceromer inlays luted with resin cement.	Thermal cycling (1000 times between 5°C and 55 °C)	μTBS
Falkensammer et al., 2014.	Human premolars	Application of a two-step self-etch adhesive system. Temporary restoration. Surface cleaning with fluoride-free pumice, airborne-particle abrasion with silicoated aluminum oxide, or calcium carbonate powder for 5 s	Prefabricated feldspathic ceramicBlocks luted with resin cement.	Saline solution at 37 °C for 24 h.	SBS
Ferreira-Filho et al., 2018.	Human third molars	Application of a one-step self-etch, a two-step self-etch, a two-step etch-and-rinse, or a three-step etch-and-rinse adhesive system.	Composite resin luted with a self-adhesive resin cement.	Distilled water at 37 °C for seven days.	μTBS
Gailani et al., 2021.	Human molars	Application of a three-step total-etch or universal adhesive system. Provisional restoration. Surface sandblasting with prophy mate cleaning powder “Calcium Carbonate”. Application and polymerization of a new adhesive layer.	Ceramic blocks luted with resin cement.	Simulated Pulp Pressure at room temperature for 24 h.	μTBS
Hayashi et al., 2019.	Human mandibular premolars	Application of a universal and a low-viscosity resin composite. Temporary restoration. Cleaning with polishing brush.	Feldspathic ceramic block luted with adhesive resin cement.	Cyclic load of 118 N over 90 cycles/min for a total of 300,000 cycles	μTBS
Hironaka et al., 2016.	Human molars	Application of a two-step self-etch adhesive system and a thin layer of adhesive resin. Temporary restoration. Surface cleaning with pumice and water.	Composite resin inlays luted with dual-polymerized resin cement.	Artificial saliva for 24 h at 37 °C	μTBS
Ishii et al., 2017.	Human molars	Application of an universal adhesive system and a low viscosity restorative composite. Temporary restoration Surface cleaning with Scotchbond Universal Etchant, 3M ESPE Application and photoactivation of Scotchbond Universal Adhesive system.	CAD/CAM onlay restorations luted with resin cement.	Distilled water at 37 °C for 24 h	μTBS
Magne et al., 2007.	Human molars	Application of 3-step etch-rinse or a 2-step self-etching adhesive system. Provisional restoration. Microairborne particle abrasion of adhesive. One coat of adhesive resin was then applied and left unpolymerized until the application of the restorative material.	Composite restoration.	Distilled water at room temperature for 24 h	μTBS
Magne et al., 2005.	Human molars	Application of a 3-step etch-and-rinse dentin bonding agent Provisional restoration. Surface cleaning with airborne-particle abrasion. Application of dentin bonding agent.	Resin composite.	Distilled water at room temperature for 24 h	μTBS
Murata et al., 2018.	Human maxillary first molars	Application of a universal adhesive system and a layer of flowable composite.	CAD/CAM ceramic onlay luted with resin cement.	Distilled water at 37 °C for 24 h	μTBS
Rigos et al., 2019.	Human third molars	Application of 3-step etch-rinse adhesive system. Air-dry and silanization for 60 s	Monolithic zirconia cylinders luted with resin cement.	Distilled water at 37 °C for 24 h	SBS
Sag et al., 2020.	Human molars	Application of a 2-step self-etching adhesive system (SE Bond; Kuraray, Tokyo, Japan). Application of a 1 mm layer of Filtek Ultimate Flowable (3M ESPE, St Paul, MN, ABD) Provisional restoration	Indirect composite and resin nanoceramic CAD/CAM blocks.	Distilled water under 15 cm water pressure for 7 days.	SBS
Sakr et al., 2021.	Human molars	Application of 3-step etch-rinse adhesive system.	Resin composite discs luted with resin cement.	Distilled water for 24 h	SBS
Santana et al., 2016.	Human molars	Application of a one-step self-etching adhesive or a two-step total-etch (Single Bond, 3M) adhesive system. Airborne particle abrasion with 50-lm aluminum oxide particles	Resin composite discs luted with resin cement.	Distilled water for 24 h	μTBS
Van den Breemer et al., 2019.	Human molars	Application of one or two layers of a 3-step etch-rinse adhesive system. The same procedures were performed with the addition of a flowable resin composite. Provisional restoration Surface cleaning with pumice rubbing (SC-P), or were tribochemically silica coated (SC-PS). A silane coupling agent was applied. The primer (Optibond FL Primer, Kerr) was then applied. A thin layer of heated (40 °C) adhesive resin was next applied.	Resin composite	0.5% chloramine T solution at 37 °C for one week.	μTBS
Van den Breemer et al., 2019. (b)	Human third molars	Application of one or two layers of a two-step self-etch adhesive system or a three-step total-etch adhesive system. Or one layer of a two-step self-etch adhesive system, or a three-step total-etch adhesive system and one layer of flowable resin composite. Temporary Restoration Cleaning using pumice or silica coating Silane Application of primer. Application of adhesive.	Composite cement	Thermocycling ×10,000 cycles between 5 °C to 55 °C	SBS

Universal adhesisve systems used: Scotchbond Universal (3M ESPE), OptiBond Universal (Kerr), Prime and Bond active universal (Dentsply), Future bond Universal (Voco), All Bond Universal (Bisco), AdheSE Universal (Ivoclar Vivadent), OneCoat 7 Universal (Coltene), Clearfil Universal Bond Quick (Kuraray Noritake Dental). Three-step total-etch adhesive systems used: Adper ScotchBond multipurpose (3M ESPE), Optibond FL (Kerr). Two-step self-etch adhesive systems used: Clearfil SE Bond (Kuraray), One Coat Bond (Coltene/Whaledent AG), AdheSE (Ivoclar Vivadent). Two-step total-etch adhesive systems used: Adper Single Bond 2 (3M ESPE), Single Bond (3M ESPE), XP Bond (Dentsply). One-step self-etch adhesive systems used: Go! (SDI), Adper Prompt L-Pop (3M ESPE), Xeno V (Dentsply), ED Primer (Kuraray). Silane coupling agent used: ESPE-SIL (3M). Resin composites used: Z100 (3M ESPE), Z350 XT (3M ESPE), Filtek Z250 (3M ESPE), Solidex (SHOFU Dental), HFO composite (Micerium). Flowable resin used: Filtek Bulk Fill Flow (3M ESPE), Clearfil Majesty ES Flow (Kuraray Noritake Dental), Filtek Supreme, Ultra Flowable Restorative (3M ESPE), Grand IO Flow (VOCO). Resin cements used: Rely X ARC (3M ESPE), RelyX U200 (3M ESPE), Variolink II cement (Ivoclar Vivadent), Rely X Unicem (3M ESPE), RelyX Ultimate (3M ESPE), PANAVIA V5 (Kuraray Noritake Dental), Panavia F 2.0 (Kuraray Noritake Dental Inc), PermaCem Dual Smartmix (DMG America). Ceramics used: Super Porcelain EX-3 (Noritake Kizai Co), Authentic (Ceranay), Vitablocs Mark II (Vita Zahnfabrik), CAD/CAM Lava™ Ultimate blocks, 3M ESPE), Vita ENAMIC (Vita), CEREC AC (Omnicam), BruxZir (Solid Zirconia). Ceromers used: Targis system (Ivoclar Vivadent).

**Table 2:** Qualitative synthesis for in vitro articles.

Study	Specimen Randomization	Single Operator	Operator Blinded	Control Group	Standardized Specimens	Failure Mode	Manufacturer’s Instructions	Sample Size Calculation	Risk of Bias
Brigagão et al., 2016.	NO	NO	NO	YES	YES	YES	YES	NO	Medium
Choi et al., 2010.	YES	NO	NO	YES	YES	YES	YES	NO	Medium
Da Silva et al., 2016.	YES	NO	NO	YES	YES	YES	YES	NO	Medium
Dalby et al., 2011.	YES	YES	NO	YES	YES	YES	YES	NO	Medium
De Carvalho et al., 2020.	YES	NO	NO	YES	YES	YES	YES	YES	Medium
Deniz et al., 2021.	YES	YES	YES	YES	YES	YES	YES	YES	Low
Duarte et al., 2009.	NO	NO	NO	YES	YES	YES	YES	NO	Medium
Falkensammer et al., 2014.	NO	NO	NO	YES	YES	YES	YES	NO	Medium
Ferreira-Filho et al., 2018.	YES	NO	NO	YES	YES	YES	YES	NO	Medium
Gailani et al., 2021.	YES	NO	NO	YES	YES	YES	YES	NO	Medium
Hayashi et al., 2019.	NO	NO	NO	YES	YES	YES	YES	NO	Medium
Hironaka et al., 2016.	NO	NO	NO	YES	YES	YES	YES	NO	Medium
Ishii et al., 2017.	NO	NO	NO	YES	YES	YES	YES	NO	Medium
Magne et al., 2007.	NO	NO	NO	YES	YES	YES	YES	YES	Medium
Magne et al., 2005.	NO	NO	NO	YES	YES	YES	YES	NO	Medium
Murata et al., 2018.	NO	NO	NO	YES	YES	YES	YES	NO	Medium
Rigos et al., 2019.	YES	NO	NO	YES	YES	YES	YES	NO	Medium
Sag et al., 2020.	YES	NO	NO	YES	YES	NO	YES	NO	Medium
Sakr et al., 2021.	YES	NO	NO	YES	YES	NO	YES	NO	Medium
Santana et al., 2016.	YES	NO	NO	YES	YES	YES	YES	NO	Medium
Van den Breemer et al., 2019.	YES	NO	NO	YES	YES	YES	YES	NO	Medium
Van den Breemer et al., 2019. (b)	YES	NO	NO	YES	YES	YES	YES	NO	Medium

**Table 3:** Search strategy used in PubMed.

#1	Immediate Dentin Sealing OR Delayed dentin sealing OR Immediate dentin sealants OR Pre-hybridization OR Resin sealing
#2	Bonding OR Bond OR Bonding efficacy OR Dental bonding OR bond strength OR bonding effectiveness OR Bonding performance OR Bond performance OR adhesive properties OR Micro-tensile strength OR microtensile strength OR Microtensile bond strength OR bonding properties OR microshear bond strength OR shear bond strength OR performance
	#1 and #2

## Data Availability

Derived data supporting the findings of this study are available from the first author (L.H.) on request.

## Supplementary Materials

The following supporting information can be downloaded at: https://www.mdpi.com/article/10.3390/gels8030175/s1, Table S1: Search strategy used in The Cochrane Library; Table S2: Search strategy used in ISI Web of Science and Scielo; Table S3: Search strategy used in Scopus; Table S4: Search strategy used in Embase.

## References

[1] Hardan L., Sidawi L., Akhundov M., Bourgi R., Ghaleb M., Dabbagh S., Sokolowski K., Cuevas-Suárez C.E., Lukomska-Szymanska M. (2021). One-Year Clinical Performance of the Fast-Modelling Bulk Technique and Composite-Up Layering Technique in Class I Cavities. Polymers.

[2] El-Banna A., Sherief D., Fawzy A.S. (2019). Resin-based dental composites for tooth filling. Advanced Dental Biomaterials.

[3] Summit J.B., Robbins J.W., Hilton T.J., Schwartz R.S. (2006). Fundamentals of Operative Dentistry. A Contemporary Approach.

[4] Roberson T.M., Heymann H.O., Swift E.J. (2006). Sturdevant’s Art and Science of Operative Dentistry.

[5] Morimoto S., Rebello de Sampaio F., Braga M., Sesma N., Özcan M. (2016). Survival rate of resin and ceramic inlays, onlays, and overlays. J. Dent. Res..

[6] D’Arcangelo C., Vanini L., Casinelli M., Frascaria M., De Angelis F., Vadini M., D’Amario M. (2015). Adhesive Cementation of Indirect Composite Inlays and Onlays: A Literature Review. Compend. Contin. Educ. Dent..

[7] Swift E.J. (2009). Critical appraisal; immediate dentin sealing for indirect bonded restorations. J. Esthet. Restor. Dent..

[8] Tetsuka N. (1993). Influence of temporary cement on dentin permeability. Jpn. J. Conserv. Dent..

[9] Paul S.J., Schärer P. (1997). Effect of provisional cements on bond strength of various adhesive bonding systems on dentin. J. Oral Rehabil..

[10] Kovalsky T., Voborna I., Ingr T., Morozova Y., Misova E., Hepova M. (2022). Immediate dentin sealing: Effect of sandblasting on the layer thickness. Bratisl. Lek. Listy..

[11] Nikaido T., Takada T., Burrow M.F., Tagami J. (1992). The early bond strength of dual cured resin cement to enamel and dentin. J. Jpn. Dent. Mater..

[12] Magne P., So W.S., Cascione D. (2007). Immediate dentin sealing supports delayed restoration placement. J. Prosthet. Dent..

[13] Magne P. (2005). Immediate dentin sealing: A fundamental procedure for indirect bonded restorations. J. Esthet. Restor. Dent..

[14] Samartzi T.K., Papalexopoulos D., Sarafianou A., Kourtis S. (2021). Immediate Dentin Sealing: A Literature Review. Clin. Cosmet. Investig. Dent..

[15] Elbishari H., Elsubeihi E.S., Alkhoujah T., Elsubeihi H.E. (2021). Substantial in-vitro and emerging clinical evidence supporting immediate dentin sealing. Jpn. Dent. Sci. Rev..

[16] Nawareg M.M., Zidan A.Z., Zhou J., Chiba A., Tagami J., Pashley D.H. (2015). Adhesive sealing of dentin surfaces in vitro: A review. Am. J. Dent..

[17] Spohr A.M., Borges G.A., Platt J.A. (2013). Thickness of immediate dentin sealing materials and its effect on the fracture load of a reinforced all-ceramic crown. Eur. J. Dent..

[18] Gresnigt M.M.M., Cune M.S., Schuitemaker J., van der Made S.A.M., Meisberger E.W., Magne P., Özcan M. (2019). Performance of ceramic laminate veneers with immediate dentine sealing: An 11 year prospective clinical trial. Dent. Mater..

[19] Hu J., Zhu Q. (2010). Effect of immediate dentin sealing on preventive treatment for postcementation hypersensitivity. Int. J. Prosthodont..

[20] Van den Breemer C., Gresnigt M., Özcan M., Kerdijk W., Cune M.S. (2019). Prospective Randomized Clinical Trial on the Survival of Lithium Disilicate Posterior Partial Crowns Bonded Using Immediate or Delayed Dentin Sealing: Short-term Results on Tooth Sensitivity and Patient Satisfaction. Oper. Dent..

[21] Van den Breemer C.R.G., Cune M.S., Özcan M., Naves L.Z., Kerdijk W., Gresnigt M.M.M. (2019). Randomized clinical trial on the survival of lithium disilicate posterior partial restorations bonded using immediate or delayed dentin sealing after 3 years of function. J. Dent..

[22] Ashy L.M., Marghalani H., Silikas N. (2020). In Vitro Evaluation of Marginal and Internal Adaptations of Ceramic Inlay Restorations Associated with Immediate vs. Delayed Dentin Sealing Techniques. Int. J. Prosthodont..

[23] Shafiei F., Aghaei T., Jowkar Z. (2020). Effect of proanthocyanidin mediated immediate and delayed dentin sealing on the strength of premolars restored with composite resin inlay. J. Clin. Exp. Dent..

[24] Maciel C.M., Souto T.C.V., Melo de Mendonça A.A., Takeshita W.M., Griza S., Silva-Concílio L.R., Baroudi K., Vitti R.P. (2021). Morphological surface analysis and tensile bond strength of the immediate dentin sealing submitted to different temporary cement removal treatments. Int. J. Adhes. Adhes..

[25] Leesungbok R., Lee S.M., Park S.J., Lee S.W., Lee D.Y., Im B.J., Ahn S.J. (2015). The effect of IDS (immediate dentin sealing) on dentin bond strength under various thermocycling periods. J. Adv. Prosthodont..

[26] Falkensammer F., Arnetzl G.V., Wildburger A., Krall C., Freudenthaler J. (2014). Influence of different conditioning methods on immediate and delayed dentin sealing. J. Prosthet. Dent..

[27] Brigagão V.C., Barreto L.F.D., Gonçalves K.A.S., Amaral M., Vitti R.P., Neves A.C.C., Silva-Concílio L.R. (2017). Effect of interim cement application on bond strength between resin cements and dentin: Immediate and delayed dentin sealing. J. Prosthet. Dent..

[28] Choi Y.S., Cho I.H. (2010). An effect of immediate dentin sealing on the shear bond strength of resin cement to porcelain restoration. J. Adv. Prosthodont..

[29] da Silva C.J.R., Gonçalves I.C.S., Botelho M.P.J., Guiraldo R.D., Lopes M.B., Gonini Júnior A. (2016). Interactions between resin-based temporary materials and immediate dentin sealing. App. Adhes. Sci..

[30] Dalby R., Ellakwa A., Millar B., Martin F.E. (2012). Influence of immediate dentin sealing on the shear bond strength of pressed ceramic luted to dentin with self-etch resin cement. Int. J. Dent..

[31] De Carvalho M.A., Lazari-Carvalho P.C., Polonial I.F., de Souza J.B., Magne P. (2021). Significance of immediate dentin sealing and flowable resin coating reinforcement for unfilled/lightly filled adhesive systems. J. Esthet. Restor. Dent..

[32] Deniz S.T., Oglakci B., Yesilirmak S.O., Dalkilic E.E. (2021). The effect of immediate dentin sealing with chlorhexidine pretreatment on the shear bond strength of dual-cure adhesive cement. Microsc. Res. Tech..

[33] Duarte S., de Freitas C.R., Saad J.R., Sadan A. (2009). The effect of immediate dentin sealing on the marginal adaptation and bond strengths of total-etch and self-etch adhesives. J. Prosthet. Dent..

[34] Ferreira-Filho R.C., Ely C., Amaral R.C., Rodrigues J.A., Roulet J.F., Cassoni A., Reis A.F. (2018). Effect of Different Adhesive Systems Used for Immediate Dentin Sealing on Bond Strength of a Self-Adhesive Resin Cement to Dentin. Oper. Dent..

[35] Gailani H.F.A., Benavides-Reyes C., Bolaños-Carmona M.V., Rosel-Gallardo E., González-Villafranca P., González-López S. (2021). Effect of Two Immediate Dentin Sealing Approaches on Bond Strength of Lava™ CAD/CAM Indirect Restoration. Materials.

[36] Hayashi K., Maeno M., Nara Y. (2019). Influence of immediate dentin sealing and temporary restoration on the bonding of CAD/CAM ceramic crown restoration. Dent. Mater. J..

[37] Hironaka N.G.L., Ubaldini A.L.M., Sato F., Giannini M., Terada R.S.S., Pascotto R.C. (2018). Influence of immediate dentin sealing and interim cementation on the adhesion of indirect restorations with dual-polymerizing resin cement. J. Prosthet. Dent..

[38] Ishii N., Maseki T., Nara Y. (2017). Bonding state of metal-free CAD/CAM onlay restoration after cyclic loading with and without immediate dentin sealing. Dent. Mater. J..

[39] Magne P., Kim T.H., Cascione D., Donovan T.E. (2005). Immediate dentin sealing improves bond strength of indirect restorations. J. Prosthet. Dent..

[40] Murata T., Maseki T., Nara Y. (2018). Effect of immediate dentin sealing applications on bonding of CAD/CAM ceramic onlay restoration. Dent. Mater. J..

[41] Rigos A.E., Dandoulaki C., Kontonasaki E., Kokoti M., Papadopoulou L., Koidis P. (2019). Effect of Immediate Dentin Sealing on the Bond Strength of Monolithic Zirconia to Human Dentin. Oper. Dent..

[42] Sag B.U., Bektas O.O. (2020). Effect of immediate dentin sealing, bonding technique, and restorative material on the bond strength of indirect restorations. Braz. Dent. Sci..

[43] Sakr O.M. (2021). Immediate Dentin Sealing versus Dentin Air Abrasion Prior to Composite Inlay luting Procedures. Med. Forum..

[44] Santana V.B., de Alexandre R.S., Rodrigues J.A., Ely C., Reis A.F. (2016). Effects of Immediate Dentin Sealing and Pulpal Pressure on Resin Cement Bond Strength and Nanoleakage. Oper. Dent..

[45] van den Breemer C., Özcan M., Cune M.S., Ayres A.A., Van Meerbeek B., Gresnigt M. (2019). Effect of Immediate Dentin Sealing and Surface Conditioning on the Microtensile Bond Strength of Resin-based Composite to Dentin. Oper. Dent..

[46] Van den Breemer C.R., Özcan M., Pols M.R., Postema A.R., Cune M.S., Gresnigt M.M. (2019). Adhesion of resin cement to dentin: Effects of adhesive promoters, immediate dentin sealing strategies, and surface conditioning. Int. J. Esthet. Dent..

[47] Qanungo A., Aras M.A., Chitre V., Mysore A., Amin B., Daswani S.R. (2016). Immediate dentin sealing for indirect bonded restorations. J. Prosthodont. Res..

[48] Magne P., Douglas W.H. (1999). Porcelain veneers: Dentin bonding optimization and biomimetic recovery of the crown. Int. J. Prosthodont..

[49] Gresnigt M.M., Cune M.S., de Roos J.G., Ozcan M. (2016). Effect of immediate and delayed dentin sealing on the fracture strength, failure type and Weilbull characteristics of lithium disilicate laminate veneers. Dent. Mater..

[50] Frankenberger R., Sindel J., Krämer N., Petschelt A. (1999). Dentin bond strength and marginal adaptation: Direct composite resins vs. ceramic inlays. Oper. Dent..

[51] Pashley E.L., Comer R.W., Simpson M.D., Horner J.A., Pashley D.H., Caughman W.F. (1992). Dentin permeability: Sealing the dentin in crown preparations. Oper. Dent..

[52] McCabe J.F., Rusby S. (1994). Dentine bonding-the effect of pre-curing the bonding resin. Br. Dent. J..

[53] Dietschi D., Herzfeld D. (1998). In vitro evaluation of marginal and in- ternal adaptation of class II resin composite restorations after thermal and occlusal stressing. Eur. J. Oral Sci..

[54] Bertschinger C., Paul S.J., Lüthy H., Schärer P. (1996). Dual application of dentin bonding agents: Effect on bond strength. Am. J. Dent..

[55] Paul S.J., Schärer P. (1997). The dual bonding technique: A modified method to improve adhesive luting procedures. Int. J. Periodontics Restor. Dent..

[56] Van den Breemer C.R.G., Buijs G.J., Cune M.S., Özcan M., Kerdijk W., Van der Made S., Gresnigt M.M.M. (2021). Prospective clinical evaluation of 765 partial glass-ceramic posterior restorations luted using photo-polymerized resin composite in conjunction with immediate dentin sealing. Clin. Oral Investig..

[57] Aggarwal V., Bhasin S.S. (2018). Application of Calcium Silicate Materials After Acid Etching May Preserve Resin-Dentin Bonds. Oper. Dent..

[58] Bourgi R., Daood U., Bijle M.N., Fawzy A., Ghaleb M., Hardan L. (2021). Reinforced Universal Adhesive by Ribose Crosslinker: A Novel Strategy in Adhesive Dentistry. Polymers.

[59] Hardan L., Bourgi R., Cuevas-Suárez C.E., Zarow M., Kharouf N., Mancino D., Villares C.F., Skaba D., Lukomska-Szymanska M. (2021). The Bond Strength and Antibacterial Activity of the Universal Dentin Bonding System: A Systematic Review and Meta-Analysis. Microorganisms.

[60] Hardan L., Bourgi R., Kharouf N., Mancino D., Zarow M., Jakubowicz N., Haikel Y., Cuevas-Suárez C.E. (2021). Bond Strength of Universal Adhesives to Dentin: A Systematic Review and Meta-Analysis. Polymers.

[61] Da Rosa L.S., Follak A.C., Lenzi T.L., Rocha R.O., Soares F.Z.M. (2018). Phosphoric Acid Containing Chlorhexidine Compromises Bonding of Universal Adhesive. J. Adhes. Dent..

[62] Malaquias P., Gutierrez M.F., Hass V., Stanislawczuk R., Bandeca M.C., Arrais C.A.G., Farago P.V., Reis A., Loguercio A.D. (2018). Two-year Effects of Chlorhexidine-containing Adhesives on the In Vitro Durability of Resin-dentin Interfaces and Modeling of Drug Release. Oper. Dent..

[63] Albuquerque M., Pegoraro M., Mattei G., Reis A., Loguercio A.D. (2008). Effect of double-application or the application of a hydrophobic layer for improved efficacy of one-step self-etch systems in enamel and dentin. Oper. Dent..

[64] Prati C., Chersoni S., Mongiorgi R., Pashley D.H. (1998). Resin-infiltrated dentin layer formation of new bonding systems. Oper. Dent..

[65] Koshiro K., Inoue S., Tanaka T., Koase K., Fujita M., Hashimoto M., Sano H. (2004). In vivo degradation of resin-dentin bonds produced by a self-etch vs. a total-etch adhesive system. Eur. J. Oral Sci..

[66] Hashimoto M., Ito S., Tay F.R., Svizero N.R., Sano H., Kaga M., Pashley D.H. (2004). Fluid move- ment across the resin-dentin interface during and after bonding. J. Dent. Res..

[67] Tay F.R., Pashley D.H. (2003). Water treeing—A potential mechanism for degradation of dentin adhesives. Am. J. Dent..

[68] Tay F.R., Pashley D.H. (2003). Have dentin adhesives become too hydrophilic?. J. Can. Dent. Assoc..

[69] Sofan E., Sofan A., Palaia G., Tenore G., Romeo U., Migliau G. (2017). Classification review of dental adhesive systems: From the IV generation to the universal type. Ann. Stomatol..

[70] Frassetto A., Breschi L., Turco G., Marchesi G., Di Lenarda R., Tay F.R., Pashley D.H., Cadenaro M. (2016). Mechanisms of degradation of the hybrid layer in adhesive dentistry and therapeutic agents to improve bond durability—A literature review. Dent. Mater..

[71] Van Meerbeek B., Yoshihara K., Van Landuyt K., Yoshida Y., Peumans M. (2020). From Buonocore’s Pioneering Acid-Etch Technique to Self-Adhering Restoratives. A Status Perspective of Rapidly Advancing Dental Adhesive Technology. J. Adhes. Dent..

[72] Stavridakis M.M., Krejci I., Magne P. (2005). Immediate dentin sealing of onlay preparations: Thickness of pre-cured Dentin Bonding Agent and effect of surface cleaning. Oper. Dent..

[73] De Goes M.F., Giannini M., Di Hipólito V., Carrilho M.R., Daronch M., Rueggeberg F.A. (2008). Microtensile bond strength of adhesive systems to dentin with or without application of an intermediate flowable resin layer. Braz. Dent. J..

[74] Breschi L., Mazzoni A., Ruggeri A., Cadenaro M., Di Lenarda R., De Stefano Dorigo E. (2008). Dental adhesion review: Aging and stability of the bonded interface. Dent. Mater..

[75] Jayasooriya P.R., Pereira P.N., Nikaido T., Tagami J. (2003). Efficacy of a resin coating on bond strengths of resin cement to dentin. J. Esthet. Restor. Dent..

[76] Van Meerbeek B., Willems G., Celis J.P., Roos J.R., Braem M., Lambrechts P., Vanherle G. (1993). Assessment by nano-indentation of the hardness and elasticity of the resin-dentin bonding area. J. Dent. Res..

[77] Roggendorf M.J., Krämer N., Appelt A., Naumann M., Frankenberger R. (2011). Marginal quality of flowable 4-mm base vs. conventionally layered resin composite. J. Dent..

[78] Hardan L., Lukomska-Szymanska M., Zarow M., Cuevas-Suárez C.E., Bourgi R., Jakubowicz N., Sokolowski K., D’Arcangelo C. (2021). One-Year Clinical Aging of Low Stress Bulk-Fill Flowable Composite in Class II Restorations: A Case Report and Literature Review. Coatings.

[79] Faggion C.M. (2012). Guidelines for reporting pre-clinical in vitro studies on dental materials. J. Evid. Based. Dent. Pract..

[80] Josic U., Sebold M., Lins R.B.E., Savovic J., Mazzitelli C., Maravic T., Mazzoni A., Breschi L. (2021). Does immediate dentin sealing influence postoperative sensitivity in teeth restored with indirect restorations? A systematic review and meta-analysis. J. Esthet. Restor. Dent..

[81] Page M.J., McKenzie J.E., Bossuyt P.M., Boutron I., Hoffmann T.C., Mulrow C.D., Shamseer L., Tetzlaff J.M., Akl E.A., Brennan S.E. (2021). The PRISMA 2020 statement: An updated guideline for reporting systematic reviews. BMJ.

[82] Bourgi R., Hardan L., Rivera-Gonzaga A., Cuevas-Suárez C.E. (2021). Effect of Warm-Air Stream for Solvent Evaporation on Bond Strength of Adhesive Systems: A Systematic Review and Meta-Analysis of in Vitro Studies. Int. J. Adhes. Adhes..

