# Supplementary material for: Immediate Dentin Sealing for Adhesive Cementation of Indirect Restorations: A Systematic Review and Meta-Analysis
Source: Gels. 2022 Mar 11;8(3):175. doi: 10.3390/gels8030175 (PMC8955250; doi:10.3390/gels8030175)
Supplement: Supplementary file 1 [file gels-08-00175-s001.zip › gels-1619497-supplementary.pdf]

Table S1. Search strategy used in The Cochrane Library.

	Search strategy
# 1	Immediate Dentin Sealing OR Delayed dentin sealing OR Immediate dentin sealants OR Pre-hybridization OR Resin sealing.
# 2	Bond OR Bonding OR Dental bonding OR Bonding efficacy OR bond strength OR Bonding performance OR bonding effectiveness OR Bond performance OR adhesive properties OR microtensile strength OR Micro-tensile strength OR bonding properties OR Microtensile bond strength OR shear bond strength OR microshear bond strength OR performance OR Clinical trials OR Controlled Clinical Trial OR Retrospective Studies OR Randomized Controlled Trial OR Prospective clinical trial OR Retrospective Study OR Prospective Studies OR Prospective Study OR Clinical Trial OR Randomized clinical trial
# 3	#1 and #2

Table S2. Search strategy used in ISI Web of Science and Scielo.

	Search strategy
# 1	TS=(Immediate Dentin Sealing OR Delayed dentin sealing OR Immediate dentin sealants OR Pre-hybridization OR Resin sealing)
# 2	TS=(Bonding OR Dental bonding OR bond strength OR adhesive properties OR microtensile strength OR Micro-tensile strength OR bonding properties OR Microtensile bond strength OR shear bond strength OR microshear bond strength OR performance OR Clinical trials OR Controlled Clinical Trial OR Retrospective Studies OR Randomized Controlled Trial OR Prospective clinical trial OR Retrospective Study OR Prospective Studies OR Prospective Study OR Clinical Trial OR Randomized clinical trial)
# 3	#1 and #2

Table S3. Search strategy used in Scopus.

	Search strategy
# 1	ALL("Immediate Dentin Sealing" OR "Delayed dentin sealing" OR "Immediate dentin sealants" OR "Pre-hybridization" OR "Resin sealing") AND TITLE-ABS-KEY("Bond" OR "Bonding" OR "Dental bonding" OR "Bonding efficacy" OR "bond strength" OR "Bonding performance" OR "bonding effectiveness" OR "Bond

	performance” OR “adhesive properties” OR “microtensile strength” OR “Micro-tensile strength” OR “bonding properties” OR “Microtensile bond strength” OR “shear bond strength” OR “microshear bond strength” OR “performance” OR "Clinical trials" OR "Controlled Clinical Trial" OR "Retrospective Studies" OR "Randomized Controlled Trial" OR "Prospective clinical trial" OR "Retrospective Study" OR "Prospective Studies" OR "Prospective Study" OR "Clinical Trial" OR "Randomized clinical trial")

Table S4. Search strategy used in Embase.

	Search strategy
# 1	‘Immediate Dentin Sealing’ OR ‘Delayed dentin sealing’ OR ‘Immediate dentin sealants’ OR ‘Pre-hybridization’ OR ‘Resin sealing’
# 2	‘Bonding’ OR ‘Dental bonding’ OR ‘bond strength’ OR ‘adhesive properties’ OR ‘microtensile strength’ OR ‘Micro-tensile strength’ OR ‘bonding properties’ OR ‘Microtensile bond strength’ OR ‘shear bond strength’ OR ‘microshear bond strength’ OR ‘performance’ OR ‘Clinical trials’ OR ‘Controlled Clinical Trial’ OR ‘Retrospective Studies’ OR ‘Randomized Controlled Trial’ OR ‘Prospective clinical trial’ OR ‘Retrospective Study’ OR ‘Prospective Studies’ OR ‘Prospective Study’ OR ‘Clinical Trial’ OR ‘Randomized clinical trial’
# 3	#1 and #2
